# Metagenomic Evaluation of Bacterial and Archaeal Diversity in the Geothermal Hot Springs of Manikaran, India

**DOI:** 10.1128/genomeA.01544-14

**Published:** 2015-02-19

**Authors:** Sonu Bhatia, Navneet Batra, Ashish Pathak, Stefan J. Green, Amit Joshi, Ashvini Chauhan

**Affiliations:** a Department of Biotechnology, GGDSD College, Chandigarh, India; b School of the Environment, Florida A&M University, Tallahassee, Florida, USA; c DNA Services Facility, University of Illinois at Chicago, Chicago, Illinois, USA; d Department of Biotechnology, SGGS College, Chandigarh, India

## Abstract

Bacterial and archaeal diversity in geothermal spring water were investigated using 16S rRNA gene amplicon metagenomic sequencing. This revealed the dominance of *Firmicutes*, *Aquificae*, and the Deinococcus-Thermus group in this thermophilic environment. A number of sequences remained taxonomically unresolved, indicating the presence of potentially novel microbes in this unique habitat.

## GENOME ANNOUNCEMENT

North India is home to several geothermal springs, presenting an opportunity to study their microbial ecology (1, 2). Of particular interest are a series of hot water springs located close to Manikaran (32°02′N, 77°21′E; elevation 1,760 m) in the northwestern Himalayas (3). This geothermal field lies in the Parbati Valley and extends in a linear zone of 1.5 km, where, sporadically, thermal springs emerge as spouts with temperatures of up to 96°C (3–5).

Previous microbial ecology studies on Manikaran springs mainly utilized culture-dependent approaches (1, 2); thus, our objective was to use metagenomics so that a comprehensive understanding of bacterial and archaeal diversity in these springs can be obtained. Replicate water samples were collected in sterilized containers from three sites at the Manikaran springs and filtered through 0.2-µm pore-size sterilized filters by vacuum filtration. The filters were stored on ice and transported to GGDSD College, Chandigarh, where genomic DNA was extracted using the Powersoil DNA Isolation Kit (MoBio Laboratories, Inc., Carlsbad, CA, USA). DNA was then amplified using a two-stage PCR approach, similar to that described previously (6). Briefly, 515F and 806R primers (7, 8), targeting the V4 variable region of bacterial and archaeal small subunit rRNA genes, were used for amplification and sequencing through adapter and bar code incorporation via a second, 8-cycle PCR employing the AccessArray Barcode Library for Illumina (Fluidigm, South San Francisco, CA, USA). Cycling conditions for the first reaction were 95°C for 5 min, followed by 28 cycles of 95°C for 30 sec, 55°C for 45 sec, and 68°C for 30 sec, with a 7-min elongation step at 68°C. The second reaction was at 95°C for 5 min, followed by 8 cycles of 95°C for 30 sec, 60°C for 45 sec, and 68°C for 30 sec, with a 7-min elongation step at 68°C. Samples were pooled and purified using solid-phase reversible immobilization implemented with AMPure XP beads. Sequencing was performed using an Illumina MiSeq Microbiology kit, with primers CS1_515F and CS2_806R and the CS2rc primer (Fluidigm) for the index read. Raw sequences were merged using CLC genomics version 7.5.1 (CLC bio, Qiagen, Boston, MA, USA) and quality trimmed (Q20) to obtain a total of 56.7 Mb of sequence data, which were analyzed using MG-RAST (9).

The Gram-positive, endospore-forming *Firmicutes* (28 to 84%) were dominant in the spring water, followed by *Aquificae* (2 to 64%) and the Deinococcus-Thermus group (1 to 18%). These phyla are mainly thermophilic and found in other extreme environments (10, 11). Bacillus megaterium, Bacillus sporothermodurans, Hydrogenobacter sp. GV4-1, Thermus* thermophiles*, and Thermus brockianus were the main bacterial species in the spring water.

*Crenarchaeota* (0.04–3%) was the main archaeal phylum, with Pyrobaculum aerophilum and P. cladifontis predominating because they are hyperthermophilic and metabolically versatile. Unlike many archaea, Pyrobaculum can thrive in microaerophilic environments by growing chemolithoautotrophically by sulfur reduction or organotrophically by sulfur respiration and fermentation, as shown by recent genome studies (12, 13).

Additionally, several bacterial and archaeal sequences remained taxonomically unresolved, indicating potentially novel microorganisms in this geothermal ecosystem. Additional metagenomics of this habitat will facilitate identification of microorganisms possessing industrially relevant traits, such as enzymes (14) and other compounds.

### Nucleotide sequence accession number.

The DNA sequences from this metagenomic project were deposited in the Sequence Read Archive under the accession number SRX792272.
